# ^68^Ga-DOTATOC PET/CT Uptake and Physiological Impairment in DIPNECH: An Exploratory Case Series

**DOI:** 10.3390/jcm15218316

**Published:** 2026-10-26

**Authors:** Anastasios Palamidas, Vasilios Tzilas, Demosthenes Bouros

**Affiliations:** 1 Pneumonology Clinic, Athens Medical Center, 15125 Athens, Greece; dbouros@med.uoa.gr; 2 Second Pneumonology Department, School of Medicine, Athens University, Attikon University Hospital, 12461 Athens, Greece; tzilasvasilios@gmail.com

**Keywords:** diffuse idiopathic pulmonary neuroendocrine cell hyperplasia, DIPNECH, ^68^Ga-DOTATOC PET/CT, somatostatin-receptor imaging, pulmonary function, exploratory case series, cryobiopsy

## Abstract

**Background/Objectives:** Diffuse idiopathic pulmonary neuroendocrine cell hyperplasia (DIPNECH) can cause respiratory symptoms and physiological impairment. We explored whether quantitative somatostatin-receptor imaging by positron emission tomography/computed tomography with gallium-68-labelled DOTATOC (^68^Ga-DOTATOC PET/CT) is associated with pulmonary physiology and nodule count in histologically confirmed DIPNECH. **Methods:** This retrospective, single-centre, hypothesis-generating series included six consecutive adults diagnosed during 2020–2025. Spearman correlations with exact permutation *p*-values and 95% confidence intervals related the maximum standardised uptake value (SUVmax) of the most avid lesion to diffusing capacity for carbon monoxide (DLCO), forced expiratory flow between 25% and 75% of forced vital capacity (FEF25–75) and the ratio of forced expiratory volume in 1 s to forced vital capacity (FEV1/FVC); index-lesion diameter and expected partial-volume recovery were examined. Analyses were exploratory and unadjusted for multiple comparisons. **Results:** All patients were women. SUVmax correlated inversely with DLCO (ρ = −0.90; *p* = 0.028) and FEF25–75 (ρ = −0.84; *p* = 0.044); the nodule-count–DLCO association was uncertain (ρ = −0.37; *p* = 0.50). Index lesions measured 3.6–4.7 mm, below the resolution of the PET system; lesion diameter was unrelated to SUVmax (ρ = −0.20), and the association with DLCO persisted after crude recovery correction (ρ = −0.66). **Conclusions:** Higher ^68^Ga-DOTATOC uptake of the most avid lesion accompanied greater physiological impairment in this six-patient series, and the differences in uptake were not explained by lesion size. Given the sample size, wide confidence intervals, multiple unadjusted comparisons, unadjusted comorbidities and sub-resolution lesions, these findings are hypothesis-generating only; they do not establish SUVmax as a marker of disease severity, an actionable threshold or a guide to biopsy, but support prospective investigation of somatostatin-receptor imaging in DIPNECH.

## 1. Introduction

Diffuse idiopathic pulmonary neuroendocrine cell hyperplasia (DIPNECH) is a rare pre-invasive pulmonary neuroendocrine disorder characterised by diffuse proliferation of pulmonary neuroendocrine cells within the bronchial and bronchiolar epithelium, with or without extension beyond the basement membrane to form tumourlets and carcinoid tumours [1,2]. It is recognised in the 2021 World Health Organization classification of thoracic tumours as a pre-invasive lesion and is considered a precursor within the spectrum of pulmonary neuroendocrine neoplasia [2,3,4]. Although increasingly recognised, DIPNECH remains substantially underdiagnosed because its clinical presentation frequently mimics asthma or chronic obstructive pulmonary disease, resulting in prolonged diagnostic delays [5,6,7,8,9].

The disease predominantly affects middle-aged and older women, particularly never-smokers or former smokers, and typically presents with chronic cough, exertional dyspnoea, and wheezing [5,6,10,11,12]. Pulmonary function testing most commonly demonstrates airflow obstruction, small-airway dysfunction, and varying degrees of impaired diffusing capacity, while high-resolution computed tomography (HRCT) characteristically shows multiple bilateral pulmonary nodules, mosaic attenuation, and expiratory air trapping [5,7,11,13]. Although the clinical course is usually indolent, a proportion of patients develop progressive fixed airflow limitation and clinically significant respiratory impairment [11].

Despite the increasing recognition of its clinicoradiological features, several clinically relevant questions remain unresolved. Histological confirmation remains the diagnostic reference standard; however, surgical lung biopsy is invasive and is often performed in older patients with impaired pulmonary reserve [10,11,14]. Consequently, no widely accepted criteria exist for determining which patients should undergo tissue confirmation or for selecting the most appropriate biopsy technique [14,15]. Whether quantitative somatostatin-receptor imaging adds information relevant to these decisions remains uncertain.

Pulmonary neuroendocrine cells express somatostatin receptors, providing the biological rationale for imaging with ^68^Ga-DOTATOC (gallium-68-labelled DOTA-Tyr^3^-octreotide, edotreotide) PET/CT [16,17]. Although somatostatin-receptor imaging is increasingly used to identify pulmonary neuroendocrine proliferations [4,14], the published experience in DIPNECH is limited to small series, and whether the intensity of uptake is related to the degree of physiological impairment has not been examined; the recent multinational series that characterised the clinical and radiological features of DIPNECH focused on HRCT and physiology rather than somatostatin-receptor imaging [18]. It is also unknown whether anatomical measures such as nodule count adequately reflect functional severity. In clinical practice the uptake of a lesion is summarised as its maximum standardised uptake value (SUVmax), and the SUVmax of the most avid lesion is the measure routinely recorded; we used this measure, for the reasons set out in Section 2.3, while recognising that uptake in a single lesion need not represent the diffuse bronchiolar component of the disease.

Therefore, we investigated a single-centre series of histologically confirmed DIPNECH patients who underwent ^68^Ga-DOTATOC PET/CT. The primary exploratory analysis examined the association between DOTATOC SUVmax and three individual pulmonary-function measures (DLCO, FEF25–75 and FEV1/FVC). Secondary exploratory analyses examined SUVmax in relation to FEV1 and FVC, the association between pulmonary nodule count and DLCO, a post hoc composite physiological index, and a descriptive split at the median SUVmax. Given the rarity of histologically confirmed disease, the series is small and all analyses are explicitly hypothesis-generating. A further aim was to formulate questions for the prospective evaluation of imaging and physiology in biopsy selection; the present study was not designed to determine biopsy indications or compare biopsy techniques.

## 2. Materials and Methods

### 2.1. Study Design and Patients

We conducted a retrospective, single-centre case series of consecutive adults with histologically confirmed DIPNECH diagnosed at the Athens Medical Center between January 2020 and December 2025. Eligible patients were adults (≥18 years) with histologically confirmed DIPNECH who had undergone ^68^Ga-DOTATOC PET/CT and complete pulmonary function testing, including DLCO, during the diagnostic work-up. Patients with neuroendocrine cell hyperplasia attributable to a secondary cause were to be excluded; none was encountered. The six patients described represent all patients with histologically confirmed DIPNECH identified at our centre during the study period, and no eligible patient was excluded. A case-series design was chosen because histologically confirmed DIPNECH is rare and no prior data link quantitative DOTATOC uptake to pulmonary physiology; the series is therefore exploratory and intended to generate preliminary estimates to inform future prospective studies. Because all patients had been referred to, and had undergone biopsy at, a centre with an interventional pulmonology service, the series is subject to selection bias, which is considered in Section 4.1. The study analysed only data and images already collected during routine clinical care. Formal ethics committee review was considered unnecessary under the institutional policy of the Athens Medical Center for retrospective case series, and no study-specific exemption determination was issued; all patients gave written informed consent for the use of their data and for publication (see Institutional Review Board Statement and Informed Consent Statement).

DIPNECH was diagnosed on histopathological demonstration of diffuse pulmonary neuroendocrine cell hyperplasia with or without tumourlets in the presence of compatible clinical, physiological and HRCT findings [1,2,15]. All specimens fulfilled the criteria proposed by Marchevsky and Walts, namely clusters of five or more neuroendocrine cells in three or more separate small airways together with three or more carcinoid tumourlets [19]; neuroendocrine differentiation was confirmed immunohistochemically (chromogranin A, synaptophysin and/or CD56). Slides were reviewed by two pathologists. Tissue confirmation was obtained by surgical lung biopsy (SLB; including one video-assisted thoracoscopic surgery [VATS] procedure) or transbronchial cryobiopsy, according to multidisciplinary clinical decision-making and routine institutional practice [14]; the biopsy route was chosen on clinical grounds, independently of the PET/CT findings, with transbronchial cryobiopsy preferred for the patients judged clinically most impaired. Histopathology was reviewed alongside the clinical and HRCT findings to distinguish DIPNECH from secondary or reactive pulmonary neuroendocrine cell hyperplasia. In the three patients with coexisting parenchymal disease (sarcoidosis, interstitial lung disease and cryptogenic organising pneumonia), the neuroendocrine cell hyperplasia and tumourlets were located in airways outside the areas affected by the comorbid process. Biopsies were taken from the largest nodules, that is, from the dominant lesions from which SUVmax was later measured. Histology showed diffuse neuroendocrine cell hyperplasia with tumourlets in all patients; the dominant nodules were tumourlets, and no carcinoid tumour was identified in any specimen, consistent with their size (≤5 mm) [2].

### 2.2. Clinical, Physiological and Radiological Assessment

Demographic characteristics, smoking history, presenting symptoms, pulmonary function, imaging findings, comorbidities and biopsy technique were extracted from the medical records.

Pulmonary function testing included spirometry before and after bronchodilator administration, with measurement of forced vital capacity (FVC), forced expiratory volume in 1 s (FEV1), the FEV1/FVC ratio and forced expiratory flow between 25% and 75% of FVC (FEF25–75), together with the diffusing capacity for carbon monoxide (DLCO). FVC, FEV1 and FEF25–75 are expressed as percentages of predicted values using the Global Lung Function Initiative (GLI) 2012 equations [20], and DLCO using the GLI 2017 equations [21]; all DLCO values were corrected for haemoglobin. Bronchodilator responsiveness was defined according to the 2022 European Respiratory Society/American Thoracic Society (ERS/ATS) technical standard as an increase in FEV1 or FVC of more than 10% of the predicted value [22]. Pulmonary function testing and PET/CT were performed within the same diagnostic work-up.

Pulmonary nodule burden was estimated from HRCT by counting discrete pulmonary nodules identified on thin-section imaging. Nodules were counted independently by two radiologists blinded to the PET/CT and pulmonary-function results; discrepancies were resolved by consensus. The presence of mosaic attenuation and of air trapping on expiratory images was also recorded. DLCO, FEF25–75, FVC and FEV1 are presented as percentages of predicted values. FEV1/FVC is the measured ratio expressed as a percentage, not a percentage of its predicted value. Pulmonary tests used Quark PFT (COSMED Srl, Albano Laziale, Italy). The system ran OMNIA software (version 2.5.3).

### 2.3. ^68^Ga-DOTATOC PET/CT

All patients underwent somatostatin-receptor imaging with ^68^Ga-DOTATOC PET/CT during the diagnostic work-up [16,17], acquired in accordance with the European Association of Nuclear Medicine (EANM) procedure guideline for somatostatin-receptor PET/CT [23]. All examinations were performed at the PET/CT Department of Athens Medical Center (Athens, Greece) on the same digital PET/CT system (Discovery MI Gen 2, GE HealthCare, Waukesha, WI, USA; silicon-photomultiplier LightBurst detectors in a four-ring configuration with a 20 cm axial field of view and time-of-flight capability) using the same protocol. Acquisition used the native Discovery MI Gen 2 software. No patient was receiving somatostatin analogue therapy at the time of imaging. Patients were not required to fast. The ^68^Ga-DOTATOC radiopharmaceutical was obtained from the National Centre for Scientific Research “Demokritos” (Agia Paraskevi, Greece). ^68^Ga-DOTATOC was injected intravenously at approximately 2 MBq/kg body weight (100–200 MBq), and acquisition started 60 min after injection. A low-dose, non-contrast CT (120 kV, automated tube-current modulation) was acquired for attenuation correction and anatomical localisation, followed by PET from the skull base to the mid-thigh at 2 min per bed position. PET data were reconstructed with the Bayesian penalised-likelihood algorithm (Q.Clear, GE HealthCare; β = 1200) incorporating time-of-flight and point-spread-function modelling, on a 256 × 256 matrix, with corrections for attenuation, scatter, random coincidences, dead time and radioactive decay. Standardised uptake values were normalised to body weight.

Images were reviewed using MIM version 7.08 (MIM Software Inc., Cleveland, OH, USA) by a nuclear medicine physician blinded to the pulmonary-function results and to the nodule counts. On fused PET/CT images, with the diagnostic HRCT available for correlation, every focal pulmonary lesion with uptake visually exceeding that of the surrounding lung parenchyma was identified, a volume of interest (VOI) enclosing each lesion was placed, and the highest SUVmax across all lesions in both lungs was recorded for analysis. For lesions abutting the hila or mediastinum, the VOI was adjusted manually to avoid spill-in from vascular or nodal activity. The long-axis diameter of the index lesion was measured on the CT component of the PET/CT in the lung window. No partial-volume correction was applied to the reported SUVmax. Because the spatial resolution of the system is approximately 4–5 mm, SUVmax is substantially underestimated in lesions smaller than two to three times this value, and the degree of underestimation depends steeply on lesion diameter below 5 mm; the implications for the present lesions are examined in Section 3.4 and Section 4.1. Diffuse parenchymal uptake was not quantified. Each measurement was made once; intra- and inter-observer reproducibility were not assessed.

The SUVmax of the single most avid lesion was used as the imaging variable for three reasons. First, it is the most widely reported and least operator-dependent somatostatin-receptor PET metric, is routinely documented in clinical reports, and could therefore be extracted retrospectively without re-segmentation [23]. Second, the diffuse bronchiolar component of DIPNECH produces low-grade uptake that is close to physiological lung background and cannot be reliably separated from parenchyma on retrospectively available images, so whole-lung measures of receptor burden (for example, the mean standardised uptake value (SUVmean) of the lung or a receptor-expressing volume analogous to metabolic tumour volume) were not obtainable. Third, we hypothesised that uptake in the most prominent neuroendocrine proliferation would index overall disease activity. This is an assumption rather than an established property of the measure: single-lesion SUVmax does not quantify diffuse bronchiolar involvement or total somatostatin-receptor burden, and its relationship to whole-lung uptake metrics requires prospective evaluation (see Section 4.1).

### 2.4. Composite Physiological Severity Index

As a supplementary post hoc summary of overall physiological impairment, we also constructed a composite physiological severity index; this was not a primary analysis.

DLCO, FEF25–75 and FEV1/FVC were standardised across the study cohort as z-scores. Their signs were reversed so that lower measurements corresponded to higher severity scores. The composite index was the arithmetic mean of the three sign-reversed z-scores. These measures represent gas transfer, small-airway function and airflow obstruction, respectively, and were equally weighted because no prior evidence supported differential weighting; this equal weighting was a pragmatic choice made for simplicity in the absence of evidence, not an evidence-based one, and alternative weightings could yield different results. FEV1 was not included because it is largely redundant with FEV1/FVC in an obstructive phenotype (in this cohort, ρ = 0.97 between the two) and is also reduced by processes unrelated to airway disease, such as respiratory-muscle weakness or obesity; including it would have double-weighted airflow obstruction. Within-cohort z-scores were calculated using the sample standard deviation.

Because the index is built from the same three measurements that were analysed individually, its association with SUVmax is not independent of theirs; it is reported only as a supplementary summary, and the individual pulmonary-function measurements remain the primary focus of the analysis.

### 2.5. Statistical Analysis

Continuous variables are presented as mean ± standard deviation (SD) or median (range), as appropriate. Categorical variables are summarised as counts and percentages.

Statistical analyses were performed in Python 3.13.16 using SciPy 1.18.1 and NumPy 2.5.3. Associations between DOTATOC SUVmax, pulmonary-function indices, pulmonary nodule count and the composite physiological severity index were assessed using Spearman’s rank correlation coefficient (ρ). Because asymptotic approximations are unreliable with six observations, two-sided exact permutation *p*-values were calculated by enumerating all 720 possible orderings. Ninety-five per cent confidence intervals (CIs) for ρ were obtained with the Fisher z transformation using the Bonett–Wright standard error [24]; with *n* = 6, these intervals are necessarily wide and are reported to make the imprecision of the estimates explicit. With six observations, the smallest attainable two-sided exact *p*-value is 0.003, and only near-perfect rank agreement (|ρ| ≥ 0.83 in the absence of ties) reaches *p* < 0.05, so a nominally significant correlation indicates near-monotonic ordering in a handful of patients rather than a precise estimate of effect size. Interpretation therefore rests on the direction and magnitude of ρ and on the width of its confidence interval rather than on statistical significance.

The primary descriptive associations were those between DOTATOC SUVmax and the individual pulmonary-function measurements (DLCO, FEF25–75 and FEV1/FVC); the post hoc composite index was examined only as a supplementary summary.

Additional analyses examined SUVmax in relation to FEV1 and FVC, and pulmonary nodule count in relation to DLCO. The latter was an exploratory anatomical comparison, not a validated negative control, and no formal comparison of correlation coefficients was planned or performed. Because comorbid lung disease could influence both uptake and physiology and could not be adjusted for, a leave-one-out sensitivity analysis was performed in which each correlation was recalculated after omitting each patient in turn. To examine whether the measured uptake of the index lesion could be separated from its size, index-lesion diameter was related to SUVmax, to the pulmonary-function measurements and to nodule count. The expected recovery of SUVmax for each index lesion was estimated for a uniform spherical lesion imaged with a three-dimensional Gaussian point-spread function of 4.5 mm full width at half maximum, using the analytical expression for the centre of a convolved sphere; a crude recovery-corrected SUVmax (measured SUVmax divided by the expected recovery) was examined descriptively, and the sensitivity of the conclusions to assumed resolutions of 4.0 and 5.0 mm was checked. These estimates are approximate and served only to judge whether lesion size alone could account for the observed differences in SUVmax.

As a purely descriptive observation, patients were also divided at the cohort median SUVmax (3.8) into three patients with lower and three with higher uptake, and group means of the physiological measurements and nodule count were summarised. Because this split was derived post hoc from the same six observations, it was not subjected to hypothesis testing, is not a validated clinical cut-off and is not presented as a threshold.

Given the exploratory nature of this study, limited sample size, and multiple comparisons performed, no adjustment for multiplicity was applied. All *p*-values were descriptive and should be interpreted as hypothesis-generating rather than confirmatory.

## 3. Results

### 3.1. Patient Characteristics

Six consecutive patients with histologically confirmed DIPNECH underwent ^68^Ga-DOTATOC PET/CT during the study period. All patients were women with a mean age of 70.3 ± 8.9 years (range 54–77 years). Four were never-smokers and two were former smokers. Dyspnoea was present in all patients, whereas cough occurred in four (67%) and wheezing in three (50%). Bronchodilator reversibility was observed in five patients (83%). Relevant comorbidities (Table 1) included obesity with obstructive sleep apnoea (case 1), sarcoidosis (case 2), interstitial lung disease with a history of malignancy (case 3), myopathy (case 4), asthma with vocal-cord paralysis (case 5) and cryptogenic organising pneumonia (case 6). Only one patient (case 5) was receiving inhaled therapy (an inhaled corticosteroid/long-acting β2-agonist combination) at the time of pulmonary function testing; no other patient was receiving inhaled or systemic respiratory medication. Mosaic attenuation and expiratory air trapping were present on HRCT in five of the six patients, the exception being the patient with interstitial lung disease (case 3).

Pulmonary physiology demonstrated a predominantly obstructive small-airway phenotype with impaired gas transfer. Mean FVC was 75.2 ± 10.1% predicted value, FEV1 61.7 ± 13.9% predicted value, FEV1/FVC 68.2 ± 11.7%, DLCO 59.8 ± 10.8% predicted value and FEF25–75 47.7 ± 31.5% predicted value. Mean pulmonary nodule burden was 9.8 ± 5.7 nodules, while mean ^68^Ga-DOTATOC SUVmax was 3.9 ± 1.1 (median 3.8; range 2.8–5.5). The index lesion measured 3.6–4.7 mm in long-axis diameter (median 4.15 mm; Table 1), below the spatial resolution of the PET system and within the size range of tumourlets as defined by the WHO classification (≤5 mm) [2]. Four patients underwent surgical lung biopsy (including one VATS procedure), whereas two underwent transbronchial cryobiopsy. Individual patient characteristics are summarised in Table 1, and descriptive statistics are presented in Table 2.

### 3.2. DOTATOC Uptake and Physiological Impairment

**Figure 1:**
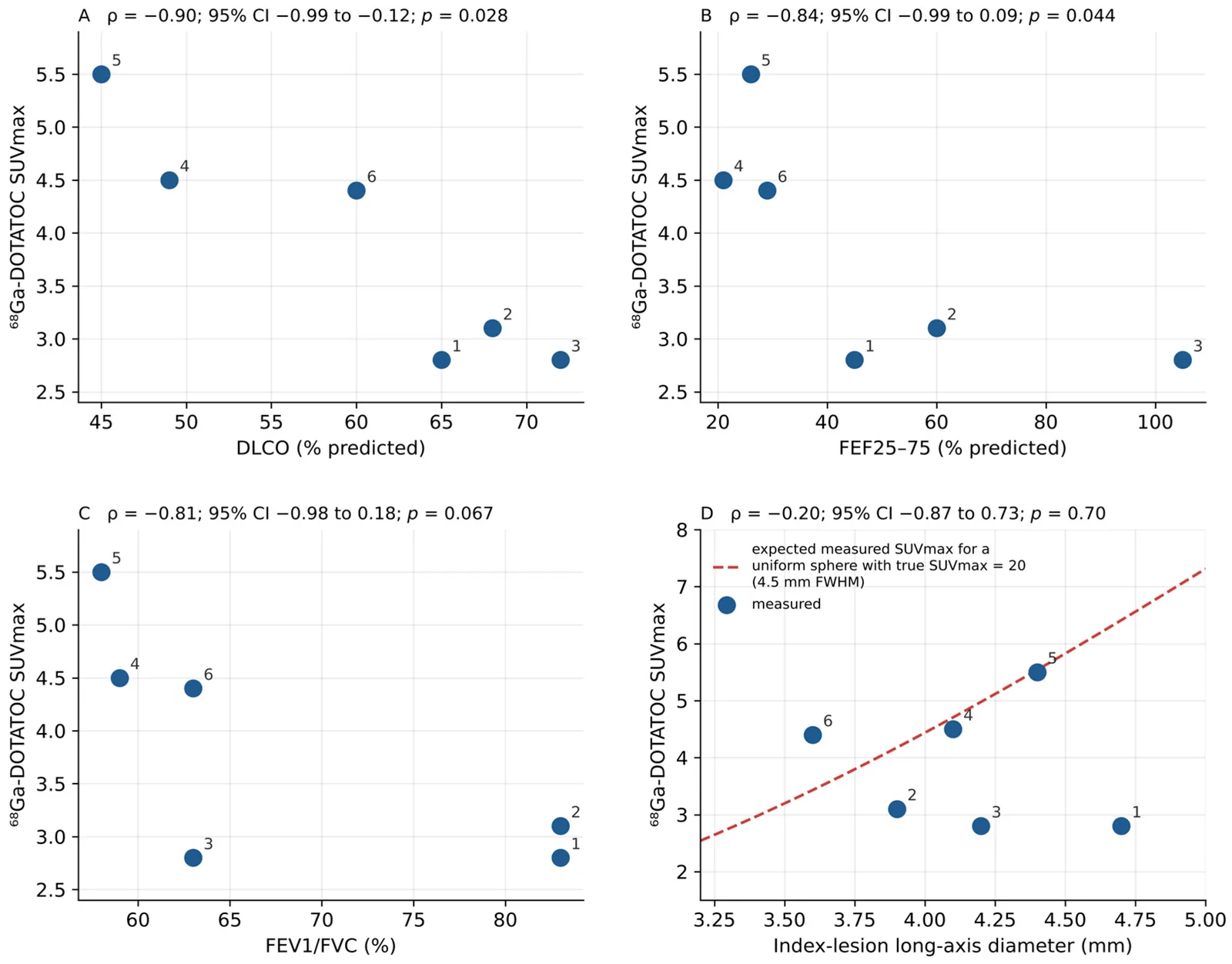
Associations of ^68^Ga-DOTATOC SUVmax with (**A**) DLCO, (**B**) FEF25–75, (**C**) FEV1/FVC and (**D**) the long-axis diameter of the index lesion in six patients with histologically confirmed DIPNECH. Each point represents one patient, labelled with the case number used in Table 1. Spearman ρ with its 95% confidence interval and exact permutation *p*-value is shown above each panel; correlations are exploratory and unadjusted for multiple comparisons. The dashed line in (**D**) is the measured SUVmax expected for a uniform spherical lesion of identical true SUVmax (20) imaged with a 4.5 mm full-width-at-half-maximum point-spread function; the measured values do not follow this curve, indicating that the differences in SUVmax between patients are not explained by lesion size, although all lesions lie in the range in which SUVmax is substantially underestimated.

DOTATOC SUVmax showed inverse associations with DLCO (ρ = −0.90; 95% CI −0.99 to −0.12; exact *p* = 0.028) and FEF25–75 (ρ = −0.84; 95% CI −0.99 to 0.09; exact *p* = 0.044) (Table 3 and Figure 1). Higher uptake was associated with lower gas-transfer and small-airway measurements in this series, but the confidence intervals extend from near-perfect inverse correlation to values compatible with a weak or absent association.

SUVmax also showed inverse but non-significant associations with FEV1/FVC (ρ = −0.81; 95% CI −0.98 to 0.18; *p* = 0.067) and FEV1 (ρ = −0.67; 95% CI −0.97 to 0.42; *p* = 0.16), and no clear association with FVC (ρ = −0.49; 95% CI −0.94 to 0.58; *p* = 0.33). All *p*-values are descriptive and unadjusted. The full leave-one-out results are given in Table S1, and the analysis script is provided as File S1. In the leave-one-out analysis, the direction of the SUVmax–DLCO association was unchanged whichever patient was omitted (ρ from −0.82 to −1.00), but with five observations, nominal significance was retained in only two of the six re-analyses; the corresponding ranges were −0.72 to −0.90 for FEF25–75 and −0.65 to −0.97 for FEV1/FVC.

As a supplementary summary, SUVmax was positively associated with the post hoc composite severity index (ρ = +0.90; 95% CI 0.12 to 0.99; *p* = 0.028; Table 3). Because the index is constructed from the same three measurements analysed individually, this association is not independent confirmation of the findings above.

Descriptively, the three patients with SUVmax above the cohort median (3.8; cases 4–6) had lower mean DLCO (51 vs. 68% predicted value), FEF25–75 (25 vs. 70% predicted value) and FEV1/FVC (60 vs. 76%) than the three below it (cases 1–3), whereas mean nodule count was similar (9.7 vs. 10.0). This split was derived from these same six observations, was not tested statistically and carries no diagnostic or biopsy-selection meaning.

### 3.3. Nodule Count and DLCO

The estimated association between nodule count and DLCO was weaker and uncertain (ρ = −0.37; 95% CI −0.92 to 0.65; *p* = 0.50). The confidence interval spans strong inverse to moderate positive correlation, so this series neither supports nor excludes a clinically relevant association. No formal comparison of correlation coefficients was performed, and the contrast between a nominally significant and a non-significant correlation should not be read as evidence that DOTATOC uptake outperforms anatomical assessment.

### 3.4. Index-Lesion Size and Partial-Volume Effects

Index-lesion diameter spanned a narrow range (3.6–4.7 mm) and was unrelated to SUVmax (ρ = −0.20; 95% CI −0.87 to 0.73; *p* = 0.70; Figure 1D), to DLCO (ρ = −0.09; *p* = 0.92), to FEF25–75 (ρ = 0.03; *p* = 1.0) or to FEV1/FVC (ρ = −0.06; *p* = 0.92); the largest index lesion (4.7 mm, case 1) had the lowest SUVmax. Because all index lesions were smaller than the spatial resolution of the system, absolute SUVmax values are substantially underestimated: for a 4.5 mm full width at half maximum, the expected recovery of SUVmax at the centre of a uniform spherical lesion ranges from approximately 0.17 at 3.6 mm to 0.32 at 4.7 mm. Partial-volume effects would therefore have compressed, not generated, the observed differences, since the least avid lesions were among the largest. A crude recovery-corrected SUVmax (measured SUVmax divided by the expected recovery) ranged from 9 to 26 and remained inversely related to DLCO (ρ = −0.66; 95% CI −0.97 to 0.43; *p* = 0.18) and FEF25–75 (ρ = −0.60; *p* = 0.24), with the same direction and magnitude for assumed resolutions of 4.0 and 5.0 mm. The differences in measured SUVmax between patients are thus not explained by lesion size, although the absolute values should not be interpreted as receptor density and the narrow range of lesion sizes limits this assessment.

## 4. Discussion

In this exploratory six-patient case series, higher ^68^Ga-DOTATOC SUVmax of the most avid pulmonary lesion was associated with lower DLCO and lower small-airway measurements. These findings are hypothesis-generating only. The index lesions measured 3.6–4.7 mm, below the resolution of the PET system, so absolute SUVmax values are substantially underestimated; lesion diameter was, however, unrelated to SUVmax, and the association with DLCO persisted after crude recovery correction, so the differences in uptake between patients are not explained by lesion size. With six observations, nominal significance requires near-perfect rank agreement, the 95% confidence intervals of every correlation extend to values compatible with weak or absent association, several correlations were examined without adjustment, and nominal significance was not retained in most of the leave-one-out re-analyses. The estimated association between nodule count and DLCO was weaker, but the small sample precludes any conclusion about the relative value of these measurements: a statistically significant association for one measure and a non-significant association for another does not establish a difference between them.

Somatostatin-receptor imaging has been described in the evaluation of pulmonary neuroendocrine proliferations [4,14,16,17]. This series explores whether quantitative uptake is associated with physiological impairment in histologically confirmed DIPNECH. Because SUVmax was obtained from the most avid pulmonary lesion, it cannot be assumed to quantify diffuse bronchiolar disease or total somatostatin-receptor burden; furthermore, the present data do not establish SUVmax as a measure of overall disease severity or of absolute receptor density because in sub-resolution lesions, the measured value is strongly attenuated by partial-volume effects. The rationale for this choice and its limitations are set out in the Methods; a prospective study should compare single-lesion SUVmax with whole-lung measures of receptor-targeted uptake.

The nodule-count–DLCO association was uncertain. Nodule count may incompletely capture bronchiolar involvement: HRCT can demonstrate nodules together with mosaic attenuation and air trapping [13], and in this series mosaic attenuation and air trapping were present in five of the six patients, so these features could not discriminate between patients and were not analysed further; their extent was not quantified. However, the present study provides no direct histological or mechanistic evidence linking receptor uptake, neuroendocrine-cell proliferation or bronchiolar remodelling to physiological impairment. Such interpretations remain hypotheses requiring targeted investigation [5,11].

Coexisting lung disease is an important potential confounder, and in this series its influence cannot be accounted for. Three patients had parenchymal comorbidity (sarcoidosis, case 2; interstitial lung disease with a history of malignancy, case 3; cryptogenic organising pneumonia, case 6), one had asthma with vocal-cord paralysis (case 5), one a myopathy (case 4) and one obesity with obstructive sleep apnoea (case 1). Interstitial lung disease, sarcoidosis and organising pneumonia can reduce DLCO; asthma and vocal-cord paralysis can reduce FEF25–75, FEV1 and FEV1/FVC; and respiratory-muscle weakness or obesity can reduce FVC and FEV1, all independently of DIPNECH. In addition, somatostatin-receptor tracer uptake is not specific to neuroendocrine cells: activated macrophages and lymphocytes express somatostatin receptors, and uptake has been documented in sarcoidosis and in fibrotic lung disease [25,26], so comorbidity could act on both variables and in either direction. The individual data do not follow a single pattern. The patients with sarcoidosis and interstitial lung disease had the two highest DLCO values and were among the three lowest SUVmax values, and the patient with interstitial lung disease had the highest FEF25–75 (105% predicted value); these conditions therefore do not account for the high-uptake/low-DLCO end of the distribution. Conversely, the lowest DLCO (45% predicted value) occurred in the patient with asthma, a condition that does not usually impair gas transfer, and the second lowest (49% predicted value) occurred in the patient with myopathy, whereas the patient with cryptogenic organising pneumonia had an intermediate DLCO (60% predicted value). In the leave-one-out analysis, the direction of the SUVmax–DLCO association did not depend on any single patient, including those with comorbid lung disease, although nominal significance did. With six patients, no adjustment, stratification or exclusion is possible, and the comorbidities cannot be assumed to bias the associations in one direction; this is a principal reason why the findings must remain hypothesis-generating. Prospective studies should record comorbid lung disease systematically and either exclude it or stratify by it.

Other explanations must also be considered. All physiological values are expressed as percentages of predicted values, which accounts for age, sex and height, and age was not correlated with SUVmax (ρ = 0.20) or with DLCO (ρ = −0.49) in this series; nevertheless, age-related changes in lung structure or tracer biodistribution cannot be excluded in a cohort aged 54–77 years. Selection is a further concern: all six patients had been referred for—and had undergone—biopsy at a centre with an interventional pulmonology service, and the biopsied population may differ from the broader DIPNECH population in both uptake and physiology. Only one patient was receiving inhaled therapy at the time of testing, so treatment effects on the physiological measurements are unlikely to explain the associations, although they cannot be quantified. The size of the index lesion was examined as an alternative explanation and did not account for the findings. All index lesions were smaller than the spatial resolution of the PET system, where the recovery of SUVmax rises steeply with diameter; however, lesion diameter spanned only 3.6–4.7 mm, was unrelated to SUVmax, and the least avid lesions were among the largest, so partial-volume effects would have compressed rather than generated the observed differences, and the association with DLCO persisted after crude recovery correction (Section 3.4; Figure 1D). The absolute SUVmax values nevertheless underestimate true uptake several-fold and should not be interpreted as receptor density; the recovery estimates are approximate; and the narrow range of lesion sizes limits the power of this assessment. Whether the uptake of the dominant tumourlet marks a more extensive or more active neuroendocrine proliferation, or whether receptor density in the diffuse bronchiolar disease relates to physiology, cannot be determined from these data.

**Figure 2:**
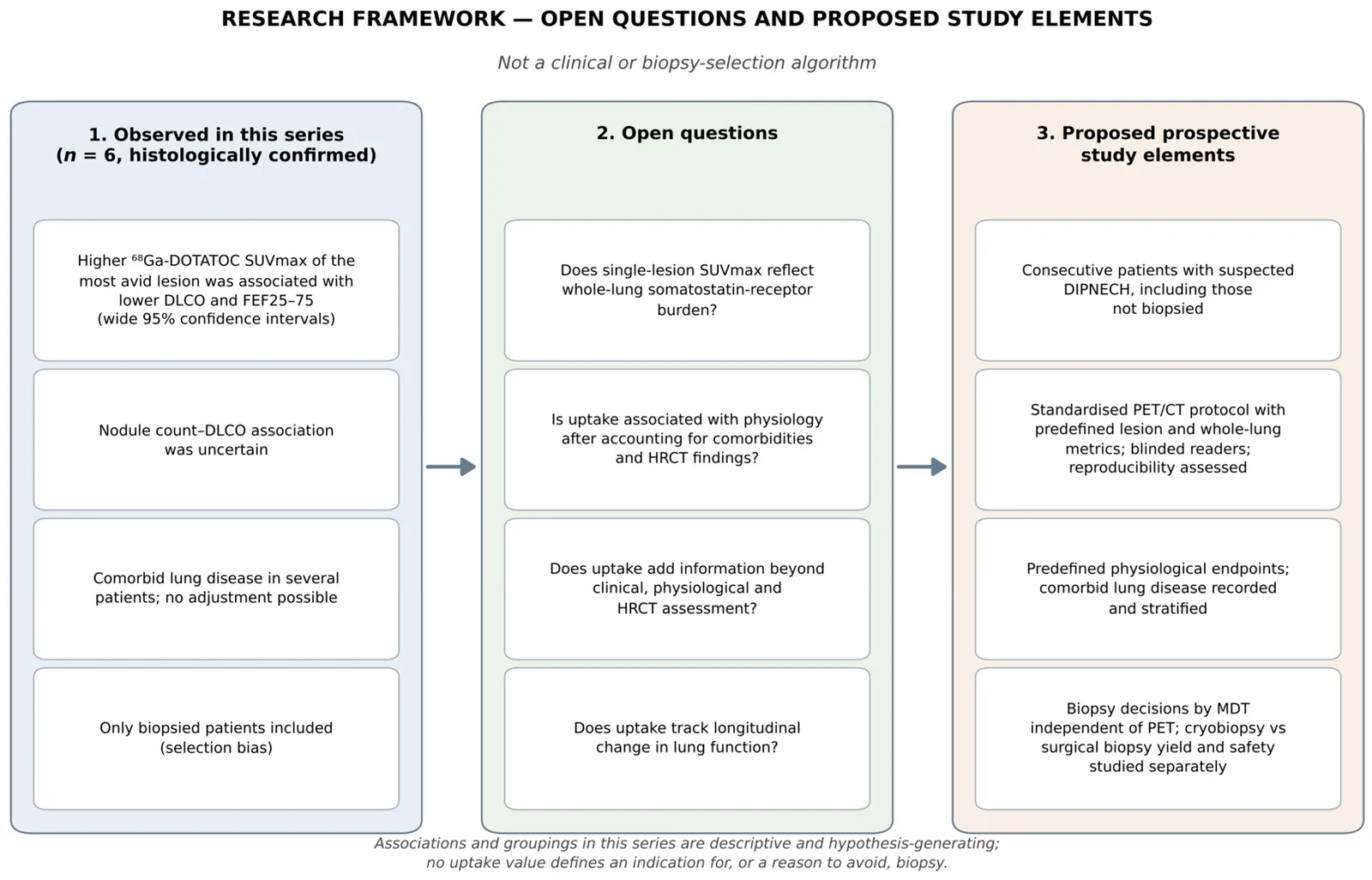
Research framework arising from this exploratory series: observations, open questions and proposed elements of a prospective study of ^68^Ga-DOTATOC uptake and pulmonary physiology in DIPNECH. The figure is not a clinical or biopsy-selection algorithm: it contains no decision branches, the six-patient series does not establish biopsy indications or an observation strategy, and no uptake value defines an indication for, or a reason to avoid, biopsy. The comparative yield and safety of cryobiopsy and surgical biopsy require separate evaluation; no first-line technique is established by these data. MDT, multidisciplinary team.

Histological confirmation can be challenging in older patients with impaired pulmonary reserve [7,10,11,14]. In this series, the biopsy route was decided by the multidisciplinary team on clinical grounds, independently of the PET/CT findings: transbronchial cryobiopsy was preferred for the two patients judged clinically most impaired, which accounts for their position in the higher-uptake group. Two cryobiopsy cases cannot establish the diagnostic yield, safety or superiority of that technique, and the series provides no information on biopsy selection, which remains a question for future research. Figure 2 therefore summarises the open questions raised by this series and the elements a prospective study would need, as a research framework rather than a clinical algorithm; it deliberately contains no decision branches, and no level of uptake or physiological impairment establishes an indication for biopsy, justifies observation without biopsy or supports cryobiopsy as first-line care.

Our findings complement rather than contradict previous reports, but the differences in sample size and design must be acknowledged. The recently published multinational series of 258 patients from 18 centres demonstrated associations between radiological abnormalities, including nodule number and mosaic attenuation, and airflow obstruction [18]; it focused on clinical, radiological and physiological characteristics rather than somatostatin-receptor imaging, and it was more than forty times larger than the present study. Our series, by contrast, comprises six histologically confirmed patients and examined the relationship between quantitative somatostatin-receptor imaging, physiological measurements and nodule count. The two studies therefore address different questions at very different levels of precision, and our observations should be read as a preliminary extension of, not a comparison with, the registry findings. Whether molecular imaging adds information beyond HRCT and physiology was not tested here and cannot be inferred from these data.

Anatomical imaging, pulmonary physiology and somatostatin-receptor imaging are intended to assess different aspects of disease expression, but an incremental diagnostic or predictive value of somatostatin-receptor imaging over HRCT and physiology has not been demonstrated. DOTATOC uptake represents somatostatin-receptor-targeted tracer accumulation and should not be equated with glucose metabolism, tumour aggressiveness or the cause of airflow limitation. Prospective studies should assess whether uptake adds information beyond clinical and HRCT findings before using it to guide diagnostic or therapeutic decisions.

### 4.1. Limitations

This retrospective single-centre series included only six histologically confirmed patients, all women, limiting precision and generalisability. With six observations, nominal statistical significance requires near-perfect rank agreement, the 95% confidence intervals of all correlations are wide, several correlations were examined without adjustment for multiplicity, and nominal significance was not retained in most of the leave-one-out re-analyses; the findings are therefore hypothesis-generating and should not be interpreted as evidence of a definite relationship. There was no non-DIPNECH comparator group, so the specificity of the observed associations for DIPNECH cannot be assessed. Three patients had parenchymal lung comorbidity (interstitial lung disease, sarcoidosis or cryptogenic organising pneumonia), one had asthma with vocal-cord paralysis, one a myopathy and one obesity with obstructive sleep apnoea. These conditions can independently influence DLCO, FEF25–75, FEV1/FVC or FVC; may also affect somatostatin-receptor tracer uptake; and did not act in a single consistent direction in the individual data. Confounding could be neither adjusted for nor characterised in this sample. Inhaled therapy was recorded (one patient) but its effect on the measurements cannot be quantified. SUVmax was taken from the single most avid lesion, measured once by a single reader and without partial-volume correction; because all index lesions were smaller than the spatial resolution of the system, absolute SUVmax values are substantially underestimated, the recovery estimates used to examine this are approximate (uniform sphere, assumed resolution, CT diameters with their own measurement error), the narrow range of lesion sizes limits the assessment of size effects, and no inference about absolute receptor density is possible. SUVmax does not quantify diffuse bronchiolar disease or total somatostatin-receptor burden, and its reproducibility and its relationship to whole-lung uptake metrics were not assessed. Nodules were counted by two blinded readers, but formal inter-rater agreement was not calculated, and the extent of mosaic attenuation and air trapping was not quantified. The composite index was developed post hoc, standardised within the same six patients and constructed from the individual physiological measurements; it is reported only as a supplementary summary and provides no independent confirmation. The descriptive median split of SUVmax was derived from this cohort and is not a clinical threshold. Because only biopsied patients were included, selection bias cannot be excluded and the study cannot determine who should undergo biopsy. The study was cross-sectional: it did not assess diagnostic accuracy, longitudinal progression, whether SUVmax predicts future physiological decline, or comparative biopsy yield and safety. These findings require prospective multicentre evaluation with standardised imaging, physiological measurements and diagnostic criteria.

### 4.2. Future Directions

The questions raised by this series are summarised in Figure 2, and several design elements would be needed to answer them. A prospective, multicentre cohort of consecutive patients with suspected DIPNECH, including those who are not biopsied and ideally linked to the international DIPNECH registry [18], would address the selection bias inherent in a biopsied series. PET/CT acquisition and reconstruction should be harmonised across sites in accordance with the EANM guideline and accreditation programmes [23], with predefined uptake metrics (SUVmax of the most avid lesion, SUVpeak and whole-lung measures of receptor-targeted uptake), documented lesion size with a predefined approach to partial-volume effects and analyses that separate uptake from lesion size, central blinded reading, and formal assessment of reproducibility. Physiological endpoints should be predefined and measured with standardised reference equations, comorbid lung disease should be recorded prospectively and used for stratification or exclusion, and a comparator group without DIPNECH should be included. Longitudinal follow-up would show whether uptake tracks or predicts physiological decline, and prespecified, adequately powered analyses with adjustment for multiplicity would replace the exploratory correlations reported here. Only such a study could determine whether somatostatin-receptor imaging adds information to HRCT and physiology and whether it has any role in selecting patients for, or the route of, biopsy.

## 5. Conclusions

In this exploratory six-patient DIPNECH series, higher ^68^Ga-DOTATOC SUVmax of the most avid pulmonary lesion was associated with greater physiological impairment, with wide confidence intervals and without adjustment for comorbid lung disease; the index lesions were smaller than the resolution of the PET system, but the differences in uptake between patients were not explained by lesion size. The association between nodule count and DLCO remained uncertain. These hypothesis-generating observations support prospective research into a possible complementary role of somatostatin-receptor imaging, with lesion size and partial-volume effects addressed by design, but they do not establish a definite relationship between uptake and physiology, superiority over anatomical measures, an SUVmax threshold or a preferred biopsy strategy. In particular, the findings do not establish SUVmax as a marker of disease severity or as a clinical threshold for biopsy or management, and the absolute SUVmax of sub-centimetre DIPNECH lesions should not be interpreted as receptor density.

## Figures and Tables

**Table 1:** Individual clinical, physiological and imaging characteristics of the six patients. (**A**) Demographics, symptoms, comorbidities and biopsy route. (**B**) Pulmonary function, bronchodilator reversibility, nodule count and uptake.

(**A**)									
**Case**	**Age** **(Years)**	**Smoking**	**Symptoms**			**Comorbidities**		**Biopsy**	
1	76	NS	Dyspnoea, cough, wheeze			Obesity, OSA		SLB	
2	54	EX	Dyspnoea			Sarcoidosis		SLB	
3	74	EX	Dyspnoea, cough			Cancer, ILD		SLB	
4	66	NS	Dyspnoea, cough, wheeze			Myopathy		SLB, VATS	
5	77	NS	Cough, dyspnoea			Asthma, vocal-cord paralysis		CRYO	
6	75	NS	Wheeze, dyspnoea			COP		CRYO	
(**B**)									
**Case**	**DLCO**	**FEF25–75**	**FVC**	**FEV1**	**FEV1/FVC**	**Reversibility**	**Nodule** **Count**	**SUVmax**	**Index Lesion (mm)**
1	65	45	72	75	83	Yes	6	2.8	4.7
2	68	60	75	78	83	Yes	20	3.1	3.9
3	72	105	82	60	63	Yes	4	2.8	4.2
4	49	21	63	49	59	No	12	4.5	4.1
5	45	26	68	43	58	Yes	10	5.5	4.4
6	60	29	91	65	63	Yes	7	4.4	3.6

(**A**) All patients were women. NS, never-smoker; EX, former smoker; OSA, obstructive sleep apnoea; ILD, interstitial lung disease; COP, cryptogenic organising pneumonia; SLB, surgical lung biopsy; VATS, video-assisted thoracoscopic surgery; CRYO, transbronchial cryobiopsy. Mosaic attenuation and expiratory air trapping were present on HRCT in cases 1, 2, 4, 5 and 6 and absent in case 3. Only case 5 was receiving inhaled therapy (inhaled corticosteroid/long-acting β2-agonist). (**B**) DLCO, diffusing capacity for carbon monoxide; FEF25–75, forced expiratory flow between 25% and 75% of FVC; FVC, forced vital capacity; FEV1, forced expiratory volume in 1 s; SUVmax, maximum standardised uptake value. DLCO, FEF25–75, FVC and FEV1 are reported as % predicted value (GLI 2012 [20] for spirometry, GLI 2017 [21] for DLCO; DLCO corrected for haemoglobin). FEV1/FVC is the measured ratio expressed as %. Index lesion, long-axis diameter of the most avid lesion on CT, in mm.

**Table 2:** Descriptive characteristics of the six-patient series.

Variable	Case Series (*n* = 6)
Age, years	70.3 ± 8.9 (median 74.5; 54–77)
Female sex	6 (100%)
Non-smoker/ex-smoker	4/2
Dyspnoea	6 (100%)
Cough	4 (67%)
Wheeze	3 (50%)
Nodule count	9.8 ± 5.7 (4–20)
FVC	75.2 ± 10.1 (63–91)
FEV1	61.7 ± 13.9 (43–78)
FEV1/FVC	68.2 ± 11.7 (58–83)
DLCO	59.8 ± 10.8 (45–72)
FEF25–75	47.7 ± 31.5 (21–105)
Bronchodilator reversibility	5 (83%)
^68^Ga-DOTATOC SUVmax	3.9 ± 1.1 (median 3.8; 2.8–5.5)
Biopsy route	SLB 3, SLB via VATS 1, cryobiopsy 2

Values are mean ± standard deviation (SD) (range) or *n* (%), unless otherwise indicated. DLCO, diffusing capacity for carbon monoxide (% predicted value); FEF25–75, forced expiratory flow between 25% and 75% of FVC (% predicted value); FEV1, forced expiratory volume in 1 s (% predicted value); FEV1/FVC, measured ratio (%); FVC, forced vital capacity (% predicted value); ^68^Ga-DOTATOC, gallium-68-labelled DOTA-Tyr^3^-octreotide; SLB, surgical lung biopsy; SUVmax, maximum standardised uptake value; VATS, video-assisted thoracoscopic surgery.

**Table 3:** Exploratory Spearman correlations in six patients, with 95% confidence intervals and exact permutation *p*-values; no adjustment for multiplicity.

Correlation	ρ (95% CI)	Exact *p*
SUVmax vs. DLCO	−0.90 (−0.99 to −0.12)	0.028
SUVmax vs. FEF25–75	−0.84 (−0.99 to 0.09)	0.044
SUVmax vs. FEV1/FVC	−0.81 (−0.98 to 0.18)	0.067
SUVmax vs. FEV1	−0.67 (−0.97 to 0.42)	0.16
Nodule count vs. DLCO	−0.37 (−0.92 to 0.65)	0.50
SUVmax vs. composite severity index (supplementary)	+0.90 (0.12 to 0.99)	0.028

CI, confidence interval (Fisher z transformation with the Bonett–Wright standard error [24]). *p*-values are two-sided exact permutation *p*-values and are descriptive. The composite index, shown for completeness only, is the mean of sign-reversed z-scores for DLCO, FEF25–75 and FEV1/FVC and is not independent of the individual measurements. DLCO, diffusing capacity for carbon monoxide; FEF25–75, forced expiratory flow between 25% and 75% of FVC; FEV1, forced expiratory volume in 1 s; FVC, forced vital capacity; SUVmax, maximum standardised uptake value.

## Data Availability

The patient-level numerical measurements supporting the reported analyses are included in Table 1. Further inquiries can be directed to the corresponding author.

## Supplementary Materials

The following supporting information can be downloaded at: https://www.mdpi.com/article/10.3390/jcm15218316/s1, Table S1: Leave-one-out sensitivity analysis of the correlations between ^68^Ga-DOTATOC SUVmax and DLCO, FEF25–75 and FEV1/FVC; File S1: Python 3.13.16 script reproducing all statistical analyses from the patient-level data in Table 1.

## Abbreviations

ATS, American Thoracic Society; CI, confidence interval; COP, cryptogenic organising pneumonia; DIPNECH, diffuse idiopathic pulmonary neuroendocrine cell hyperplasia; DLCO, diffusing capacity for carbon monoxide; DOTATOC, DOTA-Tyr^3^-octreotide (edotreotide); EANM, European Association of Nuclear Medicine; ERS, European Respiratory Society; FEF25–75, forced expiratory flow between 25% and 75% of FVC; FEV1, forced expiratory volume in 1 s; FVC, forced vital capacity; GLI, Global Lung Function Initiative; HRCT, high-resolution computed tomography; ILD, interstitial lung disease; MDT, multidisciplinary team; OSA, obstructive sleep apnoea; PET/CT, positron emission tomography/computed tomography; SD, standard deviation; SLB, surgical lung biopsy; SUVmax, maximum standardised uptake value; SUVmean, mean standardised uptake value; VATS, video-assisted thoracoscopic surgery; VOI, volume of interest.
