# Supplementary material for: 68Ga-DOTATOC PET/CT Uptake and Physiological Impairment in DIPNECH: An Exploratory Case Series
Source: J Clin Med. 2026 Oct 26;15(21):8316. doi: 10.3390/jcm15218316 (PMC13763886; doi:10.3390/jcm15218316)
Supplement: Supplementary file 1 [file jcm-15-08316-s001.zip › jcm-4581897-supplementary.pdf]

Supplementary Materials

^{68}Ga-DOTATOC PET/CT Uptake and Physiological Impairment in DIPNECH: An Exploratory Case Series

Anastasios Palamidas, Vasilios Tzilas and Demosthenes Bouros — Journal of Clinical Medicine, manuscript jcm-4581897

Table S1. Leave-one-out sensitivity analysis of the correlations between ^{68}Ga-DOTATOC SUVmax and DLCO, FEF25–75 and FEV1/FVC.

Spearman ρ (two-sided exact permutation *p*-value in parentheses) between ^{68}Ga-DOTATOC SUVmax of the most avid pulmonary lesion and each pulmonary-function measurement, recalculated after omitting each patient in turn ($n = 5$ in each re-analysis; exact *p*-values enumerate all 120 orderings). Case numbers refer to Table 1 of the main text.

Correlation	Case 1 omitted	Case 2 omitted	Case 3 omitted	Case 4 omitted	Case 5 omitted	Case 6 omitted
SUVmax vs DLCO	−1.00 (0.017)	−0.97 (0.033)	−0.90 (0.083)	−0.82 (0.133)	−0.82 (0.133)	−0.82 (0.133)
SUVmax vs FEF25–75	−0.90 (0.083)	−0.87 (0.100)	−0.80 (0.133)	−0.82 (0.133)	−0.82 (0.133)	−0.72 (0.167)
SUVmax vs FEV1/FVC	−0.82 (0.133)	−0.92 (0.067)	−0.97 (0.033)	−0.65 (0.333)	−0.65 (0.333)	−0.76 (0.183)

The direction of every association was unchanged whichever patient was omitted. With five observations, nominal significance ($p < 0.05$) was retained in two of six re-analyses for DLCO, in none for FEF25–75 and in one for FEV1/FVC. Omitting any of the three higher-uptake patients (cases 4–6) produced the weakest estimates for DLCO. DLCO, diffusing capacity for carbon monoxide; FEF25–75, forced expiratory flow between 25% and 75% of forced vital capacity; FEV1/FVC, ratio of forced expiratory volume in 1 s to forced vital capacity; SUVmax, maximum standardised uptake value.

File S1. Python 3.13.16 script reproducing all statistical analyses from the patient-level data in Table 1.

File_S1_analysis_script.py is a self-contained Python script (Python 3.13.16; SciPy 1.18.1; NumPy 2.5.3) containing the patient-level data of Table 1 and reproducing the descriptive statistics (Table 2), the Spearman correlations with two-sided exact permutation *p*-values and Bonett–Wright 95% confidence intervals (Table 3 and Figure 1), the post hoc composite index, the leave-one-out analysis (Table S1), the index-lesion size and partial-volume analysis (Section 3.4, including the sensitivity of the recovery-corrected SUVmax to the assumed resolution) and the descriptive median split reported in Section 3.2.
